# Reframing Aging in Contemporary Politics: Building Momentum

**DOI:** 10.1093/geroni/igaa057.2370

**Published:** 2020-12-16

**Authors:** Patricia D'Antonio

**Affiliations:** The Gerontological Society of America, Washington, District of Columbia, United States

## Abstract

Changing American culture is challenging and changing attitudes and behaviors around the universal experience of aging especially so. Unless the field of advocates who care about aging issues cultivates a more visible, more informed conversation on older people, it will remain difficult to advance the systemic changes needed to adjust to a society with increased and increasing longevity. Advocates will need to be vigilant to avoid cueing negative attitudes towards aging and aging policies. The Reframing Aging Initiative is a long-term, social change endeavor designed to improve the public’s understanding of what aging means and the many contributions older people bring to society. Using evidence-based research, the initiative seeks to teach advocates how to tell an effective story about aging that will promote positive perceptions of aging and reduce ageism. The time to change the conversation is now.

